# Mood by microbe: towards clinical translation

**DOI:** 10.1186/s13073-016-0292-1

**Published:** 2016-04-06

**Authors:** Timothy G. Dinan, John F. Cryan

**Affiliations:** Department of Psychiatry and Neurobehavioural Science, University College Cork, Cork, T12 YN60 Ireland; APC Microbiome Institute, University College Cork, Cork, T12 YN60 Ireland; Department of Anatomy and Neuroscience, University College Cork, Cork, T12 YN60 Ireland

## Abstract

There is a growing realization that the gut–brain axis plays a key role in maintaining brain health and the stress response. Recently, the gut microbiota has emerged as a master regulator of this axis. Thus, opportunities to exploit the microbiome to treat stress-related psychiatric disorders are materializing. Clinical validation of such strategies is now warranted.

*“All disease begins in the gut.”*

Hippocrates

The only effective pharmacological therapies developed so far for the treatment of common psychiatric disorders target the monoaminergic systems within the brain. The paradigm giving rise to such therapies dates back to the 1950s, and efforts by the pharmaceutical industry to develop therapies based on alternative paradigms have proved relatively fruitless. Over the past decade another paradigm has begun to emerge, whereby there is a growing realization that the gut–brain axis, the bidirectional communication between the digestive tract and the brain, plays a key role in maintaining brain health and the stress response. More recently, the gut microbiota has emerged as a master regulator of this axis. Indeed, preclinical studies have shown that the microbiome is key to normal neurodevelopment and behavior [1, 2], raising the potential of targeting this microbiota–gut–brain axis in the development of novel psychotropics [3]. This approach offers a promising new avenue for treating psychiatric conditions such as major depression or anxiety disorders.

## Brain–gut microbiota axis

It has been postulated that the brain–gut–microbiota axis plays a fundamental role in stress-related mental illnesses [2]. The total mass of bacteria within the intestine is approximately the same as that of the human brain, and these bacteria have a highly rich and complex biochemistry, comprising many more cells than the total number of human cells. It is estimated that this ecosystem has in excess of 1000 species and 7000 strains, but a definition of what exactly constitutes a healthy optimal microbiota is missing. At the genomic level, the global human gene complement is outnumbered by a factor of at least 100 when compared to the gut microbiome [3]. Increasing evidence indicates that the microbiota exerts a profound influence on brain physiology and ultimately on behavior, including response to stress. Further investigation is needed to understand fully how gut microbes influence the brain. Many mechanisms have been shown to be involved in this bidirectional pathway, including the vagus nerve, immune activation, and production of microbial metabolites and neurometabolites such as short chain fatty acids, vitamins and neurotransmitters. Most of the common neurotransmitters in the human brain such as GABA, 5-HT and other monoamines can be produced by bacteria, the implication of which is only slowly being unraveled.

How fundamental are gut microbes for brain function? Studies from a number of research groups in Canada, Sweden and Ireland have shown that, in germ-free animals, the brain fails to develop normally in the absence of the gut microbiome. Moreover, fundamental brain processes such as myelination, adult neurogenesis and microglia activation have also shown to be critically dependent on microbiota composition. Bercik and colleagues [4] showed that it is possible to transfer behavioral traits between mouse strains using fecal microbiota transplantation: a transplant from an anxious mouse produces an anxious phenotype while a transplant from a non-anxious mouse produces a non-anxious phenotype. They also found that transplantation alters brain chemistry in recipient ex-germ-free mice, suggesting that fecal microbiota transplantation could be used as a therapeutic avenue for disorders such as depression or anxiety.

## Depression and the microbiota

Major depression is a highly prevalent, debilitating stress-related disorder and is recognized globally as one of the significant causes of disability, with considerable social consequences. The most consistently demonstrated abnormality in depressed patients is hypothalamic–pituitary–adrenal (HPA) axis dysregulation, manifested as elevated cortisol and corticotropin releasing factor (CRF). Furthermore, significant increases in the plasma concentrations of pro-inflammatory cytokines are usually observed.

Microbes exert a major influence on both the HPA axis and on the immune system, compounding the link between the microbiota and the stress response. Sudo and colleagues [5] were the first to demonstrate that germ-free mice who grew up in a sterile environment have an exaggerated HPA axis response to an acute stressor. It is noteworthy that this enhanced responsivity of the HPA axis can be reversed by monoassociation with a single bacterial strain, in this case *Bifidobacterium infantis*. Published studies in rodents indicate that treatment with this probiotic does impact on central neurotransmitter functioning.

A number of years ago, together with Bienenstock and colleagues at MacMaster University [6], we examined the impact of a *Lactobacillus rhamnosus* strain (JB-1) on anxiety- and antidepressant-related behavior, in addition to neurochemical changes in mice. *L. rhamnosus*-treated animals had lower levels of anxiety on a variety of behavioral measures, which was concomitant with alterations in the expression of both GABA_A_ and GABA_B_ receptors across a variety of brain regions studied. Yet the question remained as to how a dietary intake of a bacterial strain could alter brain and behavior. One possible route of communication is via the vagus nerve. To test this, animals underwent vagotomy or sham surgery and were subsequently treated with *L. rhamnosus* or an inactive control broth. Indeed, vagotomy prevented the behavioral and neurochemical effects of the potential probiotic strain, suggesting that *L. rhamnosus* could serve as a potential antidepressant/anxiolytic via its (or one of its metabolites) effects on the vagus nerve. Studies are currently underway to examine the effect of this microbe on stress responses in humans; preliminary results should be available shortly.

In what is the largest study of the microbiome in major depressive disorders to date, Jiang and colleagues [7] analyzed fecal samples from 46 patients with major depression and 30 healthy controls. The authors showed that patients with depression could be stratified according to their microbiome; acutely depressed patients had higher levels of Bacteroidetes, Proteobacteria and Actinobacteria, whereas levels of Firmicutes were significantly reduced. A negative correlation was observed between Faecalibacterium and the severity of depressive symptoms. This study needs replication and further additional questions need to be answered. Are there microbes that confer a resilience against depression and are there microbes that have a melancholic impact? Assuming microbes influence mood, through which mechanisms do they produce their effects?

## Psychobiotics

Live bacteria that have a positive mental health benefit have been defined as psychobiotics [1]. Several recent studies in healthy subjects suggest that certain bacterial strains have psychobiotic activity (see [8]). However, there is a need for much more extensive placebo-controlled studies to be carried out in both healthy volunteers and especially clinical populations. Moreover, mechanistic studies focusing on brain activity patterns are required. In this vein we found that *Bifidobacterium longum* strain 1714 attenuated stress responses and enhanced cognition in healthy subjects while altering electroencephalographic activity. Mayer’s group at the University of California, Los Angeles (UCLA) found that a cocktail of bacteria produced significant changes in central physiology, as measured by altered activity in functional magnetic resonance imaging (MRI) [9].

Until very recently, the literature on psychobiotics was dominated by preclinical studies with little or no effort at translation into humans. Fortunately, this situation is now changing, with several human studies being undertaken (see [8]). However, it may take a cultural change for the food and probiotic industry to carry out the level of investment required for such clinical trials to prove efficacy. Moreover, preclinical efforts focused at dissociating the mechanisms of action of individual bacterial strains should also be increased. Rational discovery of psychobiotics will require the identification of potential therapeutic targets, be it microbial metabolites or their effectors at the receptor or cellular level of individual or a consortium of strains.

## Antimicrobials

Psychobiotics enable an increase in the level of “good” bacteria within the intestine and offer a potentially safe approach to treating stress-related conditions. Alternatively, the elimination of certain microbes using selective antimicrobial agents may have a positive mental health benefit in some individuals. Preclinical studies and initial patient-based studies indicate the potential of the antibiotic minocycline as an antidepressant [10]. Minocycline impacts on both Gram-positive and Gram-negative bacteria, although it also exerts an influence on immune mechanisms. It is tempting to speculate that its action as an antibiotic produces an antidepressant effect, at least in some patients.

## Future

Only time will tell if the brain–gut–microbiota axis proves a fruitful target for novel antidepressant development. It seems likely that psychobiotics have a role to play in the management of mild depression and anxiety states. Although animal studies have been, and continue to be, essential in deciphering the mechanisms underlying potential psychobiotic effects, we now await future translation into human clinical investigations and the results of large-scale placebo-controlled trials.

## Abbreviations

HPA: hypothalamic–pituitary–adrenal
UCLA: University of California, Los Angeles
